# The self-perceived needs of adolescents with suicidal behaviour: a scoping review

**DOI:** 10.1007/s00787-023-02342-1

**Published:** 2023-12-26

**Authors:** Milou Looijmans, Diana van Bergen, Arne Popma, Nikki van Eijk, Saskia Mérelle, Sisco van Veen, Keith Hawton, Renske Gilissen

**Affiliations:** 1 Research Department, 113 Suicide Prevention, Amsterdam, The Netherlands; 2 Child and Adolescent Psychiatry & Psychosocial Care, Amsterdam UMC, Amsterdam, The Netherlands; 3 Faculty of Pedagogical and Educational Sciences, University of Groningen, Groningen, The Netherlands; 4 Department of Psychiatry, Amsterdam UMC, Amsterdam, The Netherlands; 5 Centre for Suicide Research, University Department of Psychiatry, Warneford Hospital, Oxford, UK; 6 Oxford Health NHS Foundation Trust, Warneford Hospital, Oxford, UK

**Keywords:** Suicide, Self-harm, Prevention, Adolescents

## Abstract

Suicidal behaviour in adolescents is a major public health problem. Much research on this issue has focused on epidemiology and risk factors for suicidal behaviour, paying less attention to the self-perceived needs of adolescents. However, to increase engagement in suicide prevention, it is important to include the views of adolescents. A scoping review was performed to identify the available empirical study findings, of any study design, related to the self-perceived needs of adolescents with suicidal behaviour. The literature databases Medline, Embase, Psycinfo, CINAHL, ERIC, Scopus, and Web of Science were searched. Twenty-nine studies from 14 predominantly Anglo countries were included in the scoping review. The review showed that girls were overrepresented and that studies predominantly used qualitative approaches. The identified needs of adolescents with suicidal behaviour can broadly be grouped into needs related to the following areas: the importance of connecting with other people; adolescents’ self-help strategies and personal growth after self-harm; mental healthcare; school or study programs; and needs related to society in relation to taboo on suicidal behaviour. Prevention of suicidal behaviour in adolescents will need to focus on development and strengthening of interventions fitting the needs of adolescents in these areas.

**Supplementary Information:**

The online version contains supplementary material available at 10.1007/s00787-023-02342-1.

## Introduction

Adolescence is a vulnerable period for the development of mental health problems and specifically suicidal thoughts and behaviour. Although suicide deaths in children are relatively uncommon, the prevalence of both suicide and suicidal thoughts and behaviours increases throughout adolescence [1]. Suicide rates have increased in several countries throughout the world in recent years [2–6] and suicide is one of the leading causes of death among adolescents worldwide [7]. In this article, we are using the term ‘adolescents with suicidal behaviour’ to refer to adolescents with (a recent history of) suicidal ideation, suicide thoughts, suicide plans, and/or self-harm [8].

Important risk factors for suicidal behaviour in adolescents include, among others, social and educational disadvantage, childhood and family adversity (including trauma as child abuse, parental divorce or death), psychopathology, and social contagion [9, 10]. Two of the most important predictors of suicide in young people are engaging in self-harm [11–14] and making a suicide plan [15, 16]. Research on the perspective of adolescents of their suicidal behaviour and their specific needs is important but appears to be scarce when compared to research focusing on risk factors, risk assessment, prediction, and interventions [17–19].

However, taking into account adolescents’ own perspectives on their needs is very important. For example, young people repeatedly stress the importance of informal support, such as family, friends, and school during the times when they feel suicidal [20, 21]. Including these viewpoints is essential to guide the direction of policy and suicide prevention [20–23]*.* The self-perceived needs of adolescents to recover or to prevent relapse into suicidal behaviour seem to have become somewhat more important in research in the last years [24–26] but as far as we are aware the findings have not been assimilated in a review.

Also, potential gaps in our knowledge are undefined. In this scoping review, we present findings from studies that report on the self-perceived needs of adolescents with suicidal behaviour and also identify gaps in the research.

## Methods

### Protocol and registration

This scoping review was performed according to the PRISMA guidelines for such reviews and preregistered with the Open Science Framework on April 28, 2022 (https://osf.io/p43bq/) [27].

### Eligibility criteria

To be included, articles needed to have included adolescents who were currently suicidal or engaged in self-harm or were so in the past. Adolescents were defined as young people aged 10–25 years, since this age range corresponds most closely to adolescent growth and development to young adulthood [28]. Studies were included if they described the needs of adolescents with suicidal behaviour according to their own opinion or expressed feelings. The term ‘needs’ could refer to all areas of life, such as healthcare, school, personal relationships, family, etc. Peer-reviewed journal articles of any type of study design were included, whereas evaluation studies of specific interventions were excluded, because adolescents’ opinions on specific treatments or interventions were beyond the scope of this review. Discussion articles that did not report new empirical research (e.g., commentaries and editorials) were excluded.

### Information sources and search

To identify potentially relevant articles, the following databases were searched up to February 18, 2022 and this search was updated on May 1, 2023: Medline, Embase, Psycinfo, CINAHL, ERIC, Scopus, and Web of Science. A librarian was consulted to effectively search the databases for the three research domains: (1) adolescents or youth; (2) self-harm and/or suicidal behaviour; (3) needs, wishes, and demands. The final search strategy for PubMed can be found in Additional file 1. The search results were exported into EndNote, and duplicates were removed by the librarian. Reference lists of relevant articles and reviews were manually searched.

### Selection of sources of evidence

All identified records were uploaded to a software program called Rayyan that facilitates the screening procedure in conducting literature reviews [29]. The selection procedure involved: (1) screening on title and abstract, and (2) full-text review. Titles and abstracts of each article were independently screened by two reviewers (ML and NvE). Disagreements during screening were resolved by discussion with a third reviewer (DvB). The same procedure was followed for the selection of the full-text articles.

### Data charting and data items

The data extraction form was developed and revised during the data collection process in Microsoft Excel by two researchers (ML and NvE). Data were extracted regarding publication characteristics (country of origin, year of publication, and authors), study characteristics (purpose, setting, methods, and results) and participant characteristics (number, age range, and type of suicidal behaviour).

### Critical appraisal

Two researchers (ML and NvE) recorded the characteristics and methodological quality of each study using the Critical Appraisal Skills Program (CASP) [30], selecting the specific checklist associated with the methodology of each particular article. A third researcher (DvB) independently checked a random subsample of 20% and the results were consistent with the first appraisal.

### Synthesis of the results

Two researchers (ML and NvE) independently listed what type of needs each study examined, and, through an iterative process and discussion with the other authors, grouped the needs into five categories and summarized the type of settings, populations, and study designs.

## Results

### Sources of evidence

**Fig. 1:**
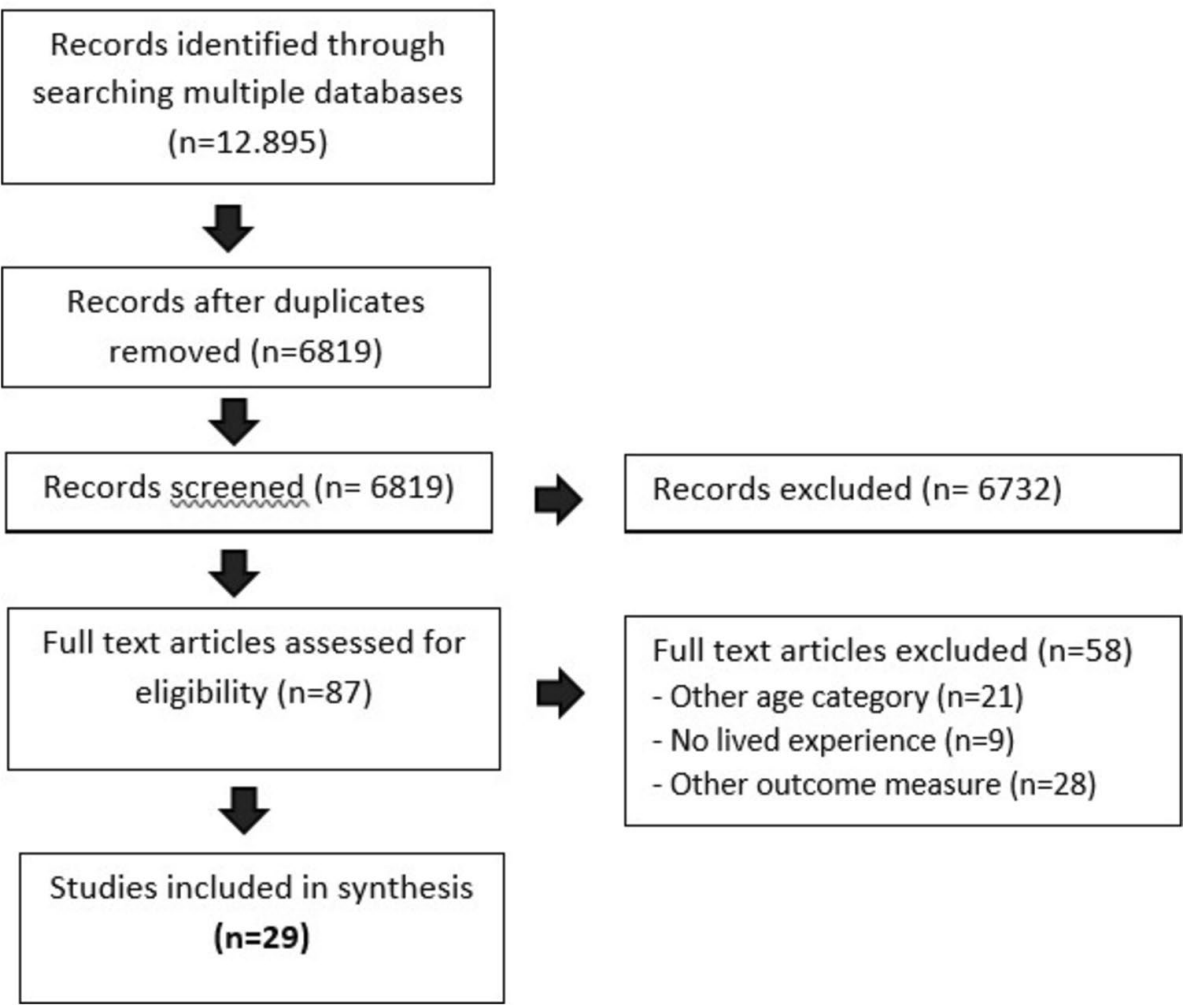
Flow diagram of the systematic search and selection process

After identifying 6819 articles, all of which included abstracts in English, 29 studies were included in the review. Of the 87 full-text articles, one article was written in French which we had translated into English. The PRISMA flow diagram in Fig. 1 gives an overview of the systematic source and selection process for the scoping review.

Eight studies were from the UK [21, 31–37], four from USA [38–41], three from Canada [42–44], two from each of Finland [45, 46], Ireland [47, 48], and Sweden [49, 50] one from each of Pakistan [51], Australia [52], New Zealand [23], South Africa [53], the Netherlands [54], Brazil [55] and Portugal [56], and one from Belgium, USA and Australia combined [57]. All studies were published between 2003 and 2023. Age of the participants ranged between 11 and 32 years at the time of study participation (all had experienced suicidality/self-harm while aged 10–25 years). A total of 1537 adolescents were included. The gender of participants was unknown for 538 adolescents. Of the remaining 999 adolescents, 166 were male and 828 female. Five adolescents defined there gender as ‘other’ (three as non-binary, one as transgender and one self-described as She/They). There were four studies that focused specifically on self-harm without intention to die [38, 41, 52, 57]. The remaining studies had included participants with an intention to die or participants who engaged in self-harm regardless of the intention or both. Seventeen studies recruited a clinical sample [23, 31, 32, 34–37, 39, 40, 43, 44, 47, 49–51, 54, 56], 11 studies recruited a non-clinical or mixed sample [21, 38, 41, 42, 45, 46, 48, 52, 53, 55, 57] and the sample of one study was not described [33].

The included studies were of variable quality, as shown in Table 1. Table 2 provides an overview of the study characteristics and the needs of adolescents with suicidal behaviour found in the reviewed literature.

**Table 1:** Critical appraisal of included studies

First author	Year	Clear statement of the aims?	Is a qualitative methodology appropriate?	Was the research design appropriate to address the aims of the research?	Was the recruitment strategy appropriate to the aims of the research?	Were the data collected in a way that addressed the research issue?	Has the relationship between researcher and participants been adequately considered?	Have ethical issues been taken into consideration?	Was the data analysis sufficiently rigorous?	Is there a clear statement of findings?	Is the research valuable?
Aspaslan	2003	Yes	Yes	Yes	Yes	Adequate	Adequate	Adequate	Can’t tell	Yes	Yes
Bergmans	2009	Yes	Yes	Yes	Yes	Can’t tell	Can’t tell	Yes	Yes	Yes	Yes
Bolger	2004	Adequate	Yes	Can’t tell	Yes	Can’t tell	Can’t tell	Can’t tell	Can’t tell	Yes	Yes
Bostik	2007	Yes	Yes	Yes	Yes	Adequate	Can’t tell	Yes	Adequate	Adequate	Yes
Doyle	2018	Yes	Yes	Adequate	Yes	Yes	No (But not needed, because it was an anonymous survey)	Yes	Yes	Adequate	Yes
Fortune	2008	Yes	Yes	Adequate	Adequate	Adequate	Can’t tell	Yes	Yes	Yes	Yes
Frost	2016	Yes	Yes	Can’t tell	Can’t tell	Can’t tell	Can’t tell	Yes	Adequate	Yes	Yes
Griffiths	2022	Yes	Yes	Adequate	Yes	Adequate	Can’t tell	Yes	Adequate	Yes	Yes
Pugnaire Gros	2012	Yes	Yes	Can’t tell	Can’t tell	Can’t tell	Can’t tell	Can’t tell	Can’t tell	Adequate	Yes
Hansson	2020	Adequate	Yes	Yes	Yes	Yes	Adequate	Yes	Adequate	Yes	Yes
Hasset	2017	Yes	Yes	Yes	Yes	Yes	Yes	Yes	Yes	Yes	Yes
Hetrick	2020	Yes	Yes	Yes	Yes	Yes	Adequate	Yes	Yes	Yes	Yes
Holland^a^	2020	Yes	Yes	Yes	Yes	Yes	Can’t tell	Yes	Yes	Yes	Yes
Idenfors	2015	Adequate	Yes	Can’t tell	Yes	Yes	Can’t tell	Yes	Adequate	Yes	Yes
Kaijadoe	2023	Yes	Yes	Yes	Yes	Yes	Can’t tell	Yes	Yes	Yes	Yes
Kelada	2016	Yes	Yes	Yes	Yes	Yes	Can’t tell	Yes	Adequate	Adequate	Yes
Kruzan	2022	Yes	Yes	Adequate	Yes	Yes	Can’t tell	Yes	Adequate	Yes	Yes
Marraccini	2021	Yes	Yes	Adequate	Yes	Yes	Can’t tell	Yes	Yes	Yes	Yes
McAndrew	2014	Yes	Yes	Yes	Can’t tell	Can’t tell	Can’t tell	Yes	Adequate	Adequate	Yes
Miettinen	2021	Yes	Yes	Yes	Yes	Adequate	Can’t tell	Yes	Adequate	Yes	Yes
Miller	2021	Yes	Yes	Yes	Yes	Yes	Can’t tell	Yes	Yes	Yes	Yes
Naz	2021	Yes	Yes	Yes	Yes	Adequate	Can’t tell	Yes	Yes	Yes	Yes
Oliveira	2022	Adequate	Yes	Adequate	Adequate	Adequate	Can’t tell	Yes	Can’t tell	Can’t tell	Yes
Rissanen	2009	Yes	Yes	Yes	Yes	Adequate	Can’t tell	Yes	Adequate	Yes	Yes
Rouski	2021	Yes	Yes	Yes	Yes	Can’t tell	Yes	Yes	Adequate	Adequate	Yes
Simoes	2021	Yes	Yes	Yes	Yes	Yes	Can’t tell	Yes	Adequate	Yes	Yes
Tillman	2018	Yes	Yes	Yes	Yes	Can’t tell	Can’t tell	Yes	Adequate	Yes	Yes
Vélez-Grau	2018	Yes	Yes	Yes	Yes	Adequate	Can’t tell	Yes	Adequate	Adequate	Yes
Wadman	2017	Yes	Yes	Yes	Yes	Yes	Can’t tell	Yes	Adequate	Adequate	Yes

^a^The article by Holland et al. [32] was not entirely qualitative in nature, but after comparing all CASP checklists, the qualitative studies version seemed most appropriate

**Table 2:** Study characteristics and the needs of adolescents with suicidal behaviour

First author, Publication year, Origin	Study purpose	Sample size	Age at time of study	Male/female/other	Type of lived experience	Context	Method	Needs
Aspaslan, 2003, South Africa	Study the experiences and needs of adolescents who have attempted suicide	N = 17	13–18	1 male/16 females	Attempted suicide	Participants were adolescents who had attempted suicide in a specific region	Semistructured interviews	**What should have been differently after attempt:** Parents: mothers more available to talk to. Hospital staff: more communication, time, attention. Social workers: more time with them alone to talk about attempt
Bergmans, 2009, Canada	Understand the transition to safer behaviors and to provide clinical suggestions for those who provide care to this population	N = 16	18–25	2 males/24 females	Attempted suicide	Participants were initially assessed for admission to the PISA intervention (a multimodal group intervention for people with recurrent suicide attempts)	Semistructured interviews	**Relevant in transition to recovery:** learning that there were choices and learning that feelings were a part of the human experience. Family, friends, and professionals were identified as important for support and education. Helpful professionals were identified as sincere, open, listening, and understanding, always up front and completely consistent. All participants identified participation in the PISA group as the single most significant experience
Bolger, 2004, Ireland	Review the clinical presentation, and AED clinical response to suicidal crisis and to carry out a follow-up	N = 31	14–20	9 males/22 females	Suicidal ideas or self-harming behaviour	Participants attended a hospital A&E department with suicidal behaviour in last 12 months	Semistructured interviews	**Support or services that would have been helpful at time of suicidal behaviour:** having someone to talk to, having a close friend or a parent to confide in, and having someone to confide in outside of the family. **Advice they would give to someone who felt like self-harming:** talk about their feelings to someone they trust. **What else is important:** having young people who have experienced mental health difficulties in the past working in any service for young people; services specifically for young people should be provided; having services that are easily accessible to young people; easy access to mental health professionals at any facility for young people
Bostik, 2007, Canada	Develop an understanding of adolescents’ perceptions of the role of attachment relationships in the process of overcoming suicidality	N = 50	13–26 (Suicidal experience 13–19)	9 males/41 females	Suicidal ideation and/or suicidal behaviour (with an intent to die)	Participants were recruited through newspaper advertisements and brochures mailed to community service agencies in two regions	Semistructured interviews	**Important in process of healing:** 1. Attachment relationships (parents, peers, extra-familial adults, spiritual connections) 2. Experiences of attachment (finding acceptance, having a permanent relationship, receiving encouragement, experiencing intimacy and closeness) 3. Changing self-perceptions (Positive interactions with others helped to see that they were important, capable people who others thought had worth and potential)
Doyle, 2018, Ireland	Identify adolescents’ attitudes about self-harm and their perspectives on preventing self-harm	N = 58^a^	15–17	NA^b^	Self-harm	Participants were recruited from 11 post-primary schools	Two items of the 6-item anonymous self-report Lifestyle and Coping survey	**What can prevent self-harm:** Talk to someone (n = 23) Focus on precipitating situation (n = 15) Professional help (n = 10) Raise awareness about mental health (n = 10) Nothing can be done (n = 6) Don’t know (n = 4) alternative coping mechanisms (n = 3)
Fortune, 2008, UK	Determine the prevalence of deliberate self-harm and factors associated with it	N = 318^c^	15–16	NA^d^	Deliberate self-harm	Participants were recruited from 41 secondary schools	Self-report questionnaires	**Top 10 what can be done to help prevent young people from feeling that they want to harm themselves:** 1. School (exams, someone to talk to, bullying, and teachers) 2.Talk/listen to them 3. Family (more love, talking, problems) 4. Formal organisations 5. Reduce barriers help-seeking (stigma, confidentially) 6. Public education 7. Friendship and peer interactions 8. Activities 9. Substances 10. Media (prevention, reduction, stigma)
Frost, 2016, Australia	Survey young people with a history of self-injury about their perceptions and preferences in regard to development of online services for self-injury	N = 457	Mean age 18 (SD = 2)	58/399	Self-injury (without intention to die)	Participants were recruited via a variety of online and offline sources	Internet survey	**Preferred sources of potential online support for self-injurious behaviour (from a list):** an online service with direct links to professionals in real time via instant messaging; peer support such as online forums and chat rooms; online self-help programs; information provided in text or fact sheets. **Most important in an online support service for self-injury:** Information; guidance; reduced isolation; online culture; facilitation of help-seeking; access; privacy
Griffiths, 2022, UK	To understand the views of children and young people (CYP), parents and staff on how staff should respond to incidents of self-harm carried out by CYP in mental health inpatient settings	N = 6^e^	13–17	1 male/5 females	Self-harm	Participants were recruited from children and young people’s mental health services	Semistructured interviews	**Important staff responses:** be genuinely interested, willing to engage in meaningful shared activities. Convey care and concern about the well-being of the CYP they are working with, and that they respond to incidents of self-harm in a validating and non-judgemental manner. CYP described needing different responses at different times, and valued staff who were able to respond flexibly to these changes
Gros, 2012, Canada	Identify the nursing interventions considered beneficial from the patient’s perspective	N = 9	15–18	2 males/7 females	Suicidality	The participants had all experienced at least one suicide risk episode in the past year, for which they followed treatment in a psychiatric care unit	Qualitative interviews and a quantitative questionnaire	**Nursing categories considered beneficial by adolescents at risk of suicide**: -Give daily support in a human and individualized way -Work together with the adolescent to manage suicide risk -Create a physical and social environment that promotes recovery
Hansson, 2020, Sweden	To increase knowledge and understanding of different aspects of life for adults with severe self-harm during adolescent inpatient stays	N = 7	29–32 (Suicidal experience 14–17)	NA	Self-harm	Participants were young adults who, when adolescents, had engaged in severe self-harm during inpatient stays in hospital	Semistructured interviews	**Positive treatment experiences:** Treatment contacts that lasted for several years, building up close relationships, were reported as valuable for improved health. Pharmacological treatment was felt to be useful when prescribed together with psychotherapy. **Recovery:** Finding a context in which other people regarded the respondents as healthy was important and also being of value to others
Hasset, 2017, UK	Explore how young men understand their journey of help-seeking and how their experiences led them to continue to seek help after initial access	N = 8	16–18	8 males	Self-harm (cutting, overdose, scratching, burning, strangulation, head banging, punching walls)	Participants were currently receiving care from CAMHS, from four National Health Service CAMHS clinics	Semistructured interviews	**Important themes in journey of help-seeking:** 1. Important role of external adult in recognizing, normalizing, and initiating help-seeking 2. Challenging and renegotiating perception of need for help and the meaning behind this need (recognition of need for help, challenging gender norms). 3. Maintaining an independent self (Choice and control, non-face-to-face methods to communicate distress). 4. Mechanisms of engagement (Developmentally sensitive approach taken by clinician, in-session techniques, shared experience)
Hetrick, 2020, New Zealand	Identify both specific triggers of the urge to self-harm, and helpful strategies to manage this urge to engage in self-harm behaviors	N = 7	18–24	2 males/5 females	Suicidal ideation and/or self-harm (irrespective of intent)	Participants were recruited from a tertiary youth mental health service and several secondary mental health services	Semistructured interviews	**Themes with regard to self -help strategies that could be used when resisting the urge to self-harm:** 1. The importance of having a diverse set of strategies 2. The importance of connecting with others 3. Changing the direct environment
Holland, 2020, UK	Answer the questions: What services do young people who self-harm find most and least helpful? Are there differences between the views of looked-after young people and those who have never been in care with regard to what they find supportive to promote recovery and reduce distress? What support would young people like which they are not currently receiving?	N = 126	11–21	20/106	Self-harm (with or without intent)	Participants were recruited from CAMHS (both inpatient and outpatient services) and Children’s Services (social care) and were identified in the community through self-harm organisations, youth clubs, secondary schools, leaflets, social media and a project website	An Audio Computer-Assisted Self-Interview (Participants hear the questions read aloud over headphones and give the appropriate response with a mouse click)	**Top 10 of most helpful services:** 1 Friends 2. Distraction techniques 3. Pets 4. Boyfriend/girlfriend 5. Exercise 6. Harm minimisation 7. Counseling 8. Teacher 9. Parent 10. Websites
Idenfors, 2015, Sweden	Explore young people’s views of professional care before first contact for DSH, and factors that influenced the establishing of contact	N = 10	17–24	4 males/6 females	Deliberate self-harm (regardless of intent)	Participants were presenting at a hospital after self-harm	Semistructured interviews	**Important factors regarding professional care:** 1. Need for many possible routes to professional care 2. The importance of immediate help 3. The importance of family and friends when overwhelmed by emotional storms 4. The importance of the perceived quality of contacts
Kaijadoe, 2023, the Netherlands	Explore how adolescents value group workers responses towards suicidal behaviour and the impact of these responses on adolescents, as well as on the group climate	N = 11	12–18	11 females	Suicidal behaviour and non-suicidal self-injury	Participants were recruited from Secure Residential Youth Care	Semistructured interviews	**Important themes in responsive reactions by group workers:** trusting the group worker, listening calmly without taking over control or being judgemental, being able to talk freely about suicidality with group worker, proximity, a hug, distraction, connectedness and showing feelings and emotions (by group workers)
Kelada, 2016, Australia Belgium USA	Understand similarities across three samples in (a) how young people define recovery from NSSI and (b) what they consider helpful approaches taken by parents and professionals to assist their recovery	N = 98	12–26^f^	23 males/75 females	Non-suicidal self-injury	Participants were recruited from secondary schools (as part of a larger study)	Australian and Belgian young people completed questionnaires, while American young people participated in interviews	**Most helpful responses from parents:** supportive and calm communication. **Most helpful responses from mental health professionals:** feeling supported, engaged, and not judged
Kruzan, 2022, USA	Understand the self-management practices of young adults who engage in NSSI, explore how they currently use technologies for self-injury self-management, and identify the ways they can envision an app-based technology supporting their self-management	N = 20	18–25	2 males/13 females/5 other	Non-suicidal self-injury	Recruited from online venues	Semistructured interviews	**To improve self-management strategies:** An app-based technology track patterns and deliver personalized suggestions for self-management
Marraccini, 2021, USA	Explore adolescent perspectives of returning to school following psychiatric hospitalization for suicide-related crises	N = 19	13–18	2 males/17 females	Hospitalization for suicide-related behaviors (i.e., suicidal ideation, suicide plans and attempts)	Participants were recruited from a psychiatric hospital	Semistructured interviews	**Recommendations to schools for improving reintegration:** 1. Supports and services (providing monitoring during the initial period return, providing a gradual return, providing support around work completion) 2. Adult relationships (connecting with adults upon return and the ways in which adults should work to get to know returning adolescents and not overly focus on academics). 3. School-wide issues (awareness and training around mental health issues)
McAndrew, 2014, UK	Elicit the narratives of young people who engage in self-harm and suicidal behaviour, in order to identify what was helpful and/or unhelpful, and what their future needs might be from a diverse range of statutory and non-statutory services	N = 7	13–17	7 females	Self-harm and/or suicidal behaviour	NA	Narrative interviews	**What would be helping:** -Service providers: being listened to; not being judged; confidentiality; trust; and being given an opportunity to talk to somebody independent of family, friends, or the school -More help within school context -Increase knowledge about self-harm to the wider population -Regular meetings with an adult confidant
Miettinen, 2021, Finland	Describe what kind of help do self-harming adolescents receive, what kind of experiences do self-harming adolescents have about help, and what kind of barriers to help have self-harming adolescents faced	N = 27	Participants did not report their age (Suicidal experience 12–22)	Both males and females (not all revealed gender)	Self-harm	Participants were recruited through the support associations websites, and closed discussions in internet support forums, in a youth rehabilitation unit, and in one hospital	Essays (N = 27) and semistructured interviews (N = 2) (2 participants did both)	**Positive impacts of treatment:** The returning ability to work through medication, the possibility of gathering oneself through medication, a helpful period in electroconvulsive therapy, and ceasing cutting due to therapy. Conversational help for depression had also been helpful for self-harm. Psychiatric in-ward treatment had helped so that the adolescent in the ward was constantly monitored, tools that made it possible to self-harm were confiscated, and feelings of being unwell or anxiety decreased in the ward. **Self-help:** Among others increasing resilience by mental growth and getting rid of guilt about illness
Miller, 2021, UK	Deepen understanding of the lived experience of female adolescent self-harm	N = 9	13–17	9 females	Self-harm	Participants were current Cambridge NHS CAMHS patients	Semistructured interviews	**Important in ‘not self-harming’:** the presence of someone who can show individual acceptance, empathy, understanding and unconditional positive regard
Naz, 2021, Pakistan	Explore the perspective of adolescents in order to identify predisposing factors of self-harm, perceived consequences, reactions of family members and their needs for services including what an acceptable psychosocial intervention would be like	N = 16	14–17	7 males/9 females	Self-harm	Participants were recruited from a public hospital	Semistructured interviews	**Suggestions regarding type of help:** -The role of emotional ventilation and having a confidant -Distraction techniques and behavioural activation in the form of activity scheduling as well as the role of learning better ways to solve problems -Adolescents can be encouraged and motivated through use of stories from lives of famous people/celebrities -Involving family members in any intervention being offered to the self-harm survivors
Oliveira, 2022, Brazil	Analyze the occurrence and characteristics of self-injury among adolescents in a public school	N = 66	11–16 (entire sample)^g^	41 males/71 females (entire sample)	Self-injurious behaviour	Students from a public school	Questionnaire with closed and open questions	**Needs from school:** Adolescents expressed a demand for a responsive and careful dialog, stating that the school needs support to discuss the topic of self-injury, as adolescents who harm themselves do not speak out
Rissanen, 2009, Finland	Describe help from the viewpoint of self-mutilating Finnish adolescents	N = 72 (62 emails and 10 interviews)	Emails: 12–21 Interviews: 15–19	62 emails: NA 10 interviews: 10 females	Self-mutilation (self-cutting)	Participants were recruited through advertising the study in four magazines targeted at adolescents, on magazine Web sites, and on the principal researcher’s own Web site	62 × written descriptions of help via email and 10 individual interviews	**Who can be a helper:** Any person who knows about the self-mutilation can be a helper (age mates, loved ones, adults (including unknown adults, health and social care professionals, teachers and counselors and parents)) **Factors that enabled help-seeking:** Becoming conscious of being in need of help, knowledge of self-mutilation as a phenomena, knowledge of the available of help for self-mutilation, caring environment, all kinds of support from friends, peers and parents **Helpful factors:** Being conscious of ones need for help, enabling early and practical intervention, intervening in adolescent problems, learning to discuss in general and especially about self-mutilation and all kinds of emotions and difficult experiences with someone, authentic caring for the adolescent, adolescents own activities
Rouski, 2021, UK	Exploring the experiences of young people who self-harm in residential care settings with a particular focus on understanding how the relational context of the setting, including staff responses, affects their experience	N = 5	14–18	2 males/3 females	Self-harm (regardless of motivation or intent)	Participants were recruited from four therapeutic residential care homes run by two residential care providers	Semistructured interviews	**Needs regarding residential care**: 1. Seeking genuine care and containment: where staff were experienced as offering genuine care, this provided participants with a sense of safety and trust in a parental figure who could notice and contain their distress. 2. The cry to be understood: young people’s need for staff to understand them, and particularly for their self-harm to be understood in the context of their lives. Where the previous theme highlights the role of staff in helping participants to manage their self-harm and cope with the underlying distress, this theme reflects their need for staff to understand their self-harm in order to help them make sense of it themselves
Simoes, 2021, Portugal	Identify the protective factors of recurrent suicidal behaviours in adolescents; To describe the family and the expectations for future involvement; To know the most important aspects of hospitalization and discuss expectations of nursing care follow-up after hospital discharge	N = 33	13–18	9 males/24 females	Suicidal behaviour	Participants were young people admitted to a child psychiatric unit and having been clinically discharged afterwards	Semistructured interviews	**Protective factors of recurrent suicidal behaviour:** family, friends and other trusted people; the self and the new strategies learnt; follow-up and health professionals **Expectations of post-discharge nursing follow-up: ‘**Keep in touch’ **Most important aspects of hospitalization:** psychological support and health professionals; occupational activities; environment; learning **Suggestions for service improvement:** Environment; interventions
Tillman, 2018, USA	Understand the lived experiences of middle school girls who have engaged in NSSI and who have received professional help for those behaviors	N = 6	Mean age 13.8 (SD = 0.41)	6 females	Non-suicidal self-injury	Participants were middle school students recruited using both a Facebook advertisement and a Facebook page designed specifically for the study	An online survey with a series of open-ended questions	**What professionals should know about NSSI:** 1. Each person is unique 2. Helping people feel comfortable in counseling 3. Understanding the reasons for self-injury
Vélez-Grau, 2018, USA	Provide an opportunity for adolescents to voice their own perspectives and for researchers and clinicians to gain an understanding of adolescents’ life experience away from the treatment setting, as well as their experience as consumers of mental health services	N = 4	15–17	1 male/3 females	Suicidal ideation and suicide attempts	Participants were recruited from a child and adolescent mental health clinic	Photovoice (method that involves focus groups and the use of cameras by participants to visually capture their reality and express their ideas through photographs)	**Wishes and expectations for clinicians and treatment:** 1. They would like clinicians to see them from different perspectives and to focus more on their strengths than on their problem-behaviors. 2. Adolescents felt it was important that clinicians heard directly from them before going to other sources of information, as was often their experience. 3. Adolescents would like to be informed and participate actively in decisions about their treatment
Wadman, 2017, UK	Gain insight into looked-after young people’s perceptions and experiences of factors related to self-harm, and of interventions and services received, in order to improve future service provision. Examine how looked-after young people make sense of the experience of self-harm and resulting supports	N = 24	14–21	4 males/20 females	Self-harm	Participants had experience of living in foster care or residential homes	Semistructured interviews	**Ways of stopping self-harm:** Participants described how they had managed to develop their own coping techniques. This reliance on self-help seemed more salient to the young people than clinical services, and was generally preferred

^a^Doyle: of the 103 participants who self-harmed, 56% (n = 58) completed the open-ended survey question

^b^Doyle: for the entire sample, the m/f ratio was 438/418

^c^Fortune: the entire sample was N = 2954

^d^Fortune: for the entire sample, the m/f ratio was 1368/1581

^e^Griffiths: the entire sample was N = 17

^f^Kelada: Australian sample (N = 48) 12–18 years, Belgium sample (N = 25) 17–19 years, American sample (N = 25) 15–26 years

^g^Oliveira: the entire sample was N = 112

### Synthesis of results

The needs of adolescents with suicidal behaviour found in the 29 articles can be categorized into the following themes: (1) connecting with others, (2) self-help strategies and personal growth, (3) mental health care, (4) school, and (5) general public.

#### Needs related to connecting with other people

The vast majority of ‘needs’ of adolescents with suicidal behaviour were related to connecting with other people [21, 23, 31–34, 36–40, 42–45, 47–51, 53, 54, 56, 57].

##### Support through connecting and recognition

In a study from New Zealand, connecting with others was described by adolescents as a key strategy to prevent self-harm. More precisely, connecting was described as helping with overcoming feelings of isolation, in dealing with negative thoughts and in increasing a sense of safety and being cared for [23]. Also, in the process from suicidality to recovery, supportive relationships with others were mentioned to be crucial [31, 42, 43]. According to the adolescents, supportive contacts can include parents, peers, family, extra-familial adults, and professionals, but also spiritual or religious connections [42, 43].

One study from the UK found that the initial recognition that help is needed often comes from connected external contacts, such as parents, friends, or schoolteachers, rather than from the adolescents themselves [31]. In another UK study, adolescents suggested that once they were engaged in regular meetings with an adult confidant (whether or not this was a professional), they eventually found the courage to reveal that they had been self-harming [33]. Although five young men (in a small qualitative sample of eight young men from the UK), indicated that they benefited from contact with an adult man; gender of the influential other was not seen as crucial in the process of recovery by these individuals [31]. Furthermore, having good accepting relationships with others also helped adolescents to feel more confident about themselves according to a Canadian study: *“I got connected with one person…there was somebody there who thought something of me, more than that I was useless and never going to amount to anything….”* [42].

In a study of adolescents in a community sample from the UK who reported self-harm, the researchers asked the question ‘What do you think can be done to help prevent young people from feeling that they want to harm themselves?’ the most frequent response was ‘listening to them and talking to them’ (27% of 318) [21]. Adolescents in this study [21] underpinned the importance of someone who is ‘there for them’ and for providing support to those who feel like harming themselves: *“People want to listen to their problems, give them confidence, be there for them, don’t let them down, show them you like them and you want to help them”.* More girls than boys mentioned aspects relating to talking, listening, and providing general support [21]. Also, with regard to self-harm, adolescents in another UK study valued the presence of someone who can show individual acceptance, empathy, understanding, and unconditional positive regard. This was evident in both outpatient and inpatient relationships [36]. In an Irish community study, in response to the question ‘what can prevent self-harm?’, the majority of adolescents who self-harmed, also answered ‘talk to someone’ (39.6% of 71). For the most part, peers were the preferred source of help as there was the belief as they would understand better and speaking to a parent or other adult would result in actions that were unfavorable for the adolescent [48]. In a study from Pakistan, adolescents with experience of self-harm mentioned emotional ventilation and having a confidant as suggestions regarding type of help that they thought can benefit adolescents who self-harm: *“I think discussion is important, those who do not do this suffocate themselves from inside, and sharing can help to relax”* [51].

According to a Finnish study, adolescents in residential care thought any person who knows about the self-harm can help. That is, friends of similar age (including fellow self-harming adolescents), and loved ones and adults (including unknown adults, health and social care professionals, teachers, counselors, and parents). Adolescents in this study mentioned contact with friends as most helpful in preventing self-harm (46% of 53) [45].

##### Needs from health care providers

Studies found that adolescents need to perceive healthcare providers as ‘sincere’, ‘open’, ‘listening’, ‘understanding’, ‘always up front’, ‘reliable’, ‘non-judgmental’, ‘confidential’, and ‘completely consistent’ [33, 43]. In a study from the UK adolescents described they need staff to be genuinely interested and willing to engage in meaningful shared activities. Staff also need to show care and concern about the well-being of the adolescents and respond to incidents of self-harm in a validating and non-judgmental manner. Adolescents in this study described needing different responses at different times, and valued staff who were able to respond flexibly to these changes [37]. In a Dutch study, adolescents in secure residential youth care described what they experienced as responsive from their group workers in relation to suicidality. Their answers centered around experiencing proximity, commitment, trust, and connection [54]. In a Swedish study, having a good connection with a professional was reported as more important to adolescents than the professional being someone from a specific profession [49]. In addition, a study from the UK showed that, when professionals offer care perceived as genuine, this provides adolescents with a sense of safety and trust in ‘a parental figure’ who could notice and contain their distress. Adolescents need professionals to understand them, and particularly to understand the reasons for and background to self-harm in the context of their lives [34]. Moreover, adolescents would like professionals to see them from different perspectives and to focus more on their strengths than on their problem behaviour. Also, a study from USA found that adolescents need professionals to hear directly from them before seeking other sources of information, for example from their parents [39].

According to a South-African study, when adolescents were asked after a suicide attempt what they would have liked to have been different in hospital care, they mainly focused on medical staff-patient relationships. The majority of adolescents would have liked ‘more communication’ from the medical staff, ‘more time and attention’ from them, and ‘more caring and supportive treatment’. In a setting where social workers were available, adolescents also mentioned that they would have had preferred more time alone with their social worker to talk about their suicide attempt [53]. Adolescents in a Canadian study expected nurses to give them daily support in a human and individualized way, to work together with the adolescent to manage suicide risk, and to create a physical and social environment that promotes recovery [44]. In a Portuguese study, the aspects adolescents who had been admitted to a child psychiatric unit with suicidal behaviour valued most about their hospitalization had to do with ‘psychological support and health professionals’ (for example ‘*knowing that people acknowledge and understand my pain’*) [56]. In a study of adolescents receiving mental health care in USA, some adolescents said that they would like to be more informed about their treatment direction and be able to participate more actively in decisions about their treatment [39]. The most helpful responses from mental health professionals mentioned by adolescents with non-suicidal self-injury in a cross-cultural study in Australia, USA and Belgium were ‘supporting’, ‘engaging’, and ‘non-judging’ responses [57]. Furthermore, according to a study from USA, mental health professionals needed to know that each young person with non-suicidal self-injury is unique; they should help adolescents to feel comfortable and need to understand the reasons for self-harm [38].

##### Peers with lived experience as source of support

Adolescents in a South-African study who attempted suicide expressed the need for a support group where they could talk to other adolescents with the same experience [53]. In a study in Canada, a specific group based on a psychosocial/psychoeducational intervention for people with recurrent suicide attempts was appreciated, because it provided an understanding peer group [43]. Irish adolescents stressed the importance of having other young people who have experienced mental health difficulties in the past working alongside professionals in any service for young people [47].

#### Needs related to adolescents’ self-help strategies and personal growth

Some of the identified needs were related to adolescents’ coping skills or personal growth on the path from suicidal behaviour to recovery [23, 31, 32, 35, 41–43, 45, 46, 51, 56].

##### Self-help strategies

A New Zealand study showed that when feeling the urge to self-harm, adolescents mentioned that how helpful a specific strategy is dependent on a range of different factors at a specific moment, such as mood, interests, and setting (e.g., home or school). Therefore, it is important for adolescents to have an understanding of their own triggers with an accompanying diverse set of strategies [23]. In a UK study, when asked about ways of stopping self-harm, adolescents in mental health care described how they had managed to develop their coping techniques. They reported activities, such as art, music, and going for walks reportedly helped them to delay and distract from self-harm [35]. Immediately moving to another place was also noted as an important self-help strategy [23]. In another UK study, reliance on self-help in stopping self-harm seemed more salient to the young people than clinical services, and was generally preferred [35]. Finally, in a study from the USA, adolescents reported a desire for an app-based technology to track patterns and deliver personalized suggestions for self-management of self-harm [41].

##### Personal growth

A Canadian study showed that adolescents who attempted suicide identified the following aspects as important on their road to recovery: learning that there are choices you can make in relation to your mental health, and that feelings are a part of the human experience. Feelings needed to be identified and tolerated to be understood and learned from:

“to be more comfortable with myself...”; “living a life that is really mine by the choosing, not by what society deems successful; or something astronomical and my first thought won’t be dying, or cutting or getting drunk or getting high, it will be to cry and move on” [43].

Getting rid of the guilt about their mental illness was mentioned by adolescents as important in a Finnish study [46], and in a Swedish study, being of value to others and finding a context in which other people regarded the respondents as healthy were reported as important to adolescents [50]. In addition, a sense of control, independence, and autonomy are important in the process to recovery, even though this sometimes conflicts with needing good and supportive relationships [31, 49].

#### Needs related to aspects of mental health care

Another theme related to specific aspects of needs related to mental health services, such as access to appropriate youth mental health care [21, 44, 46, 47, 49, 50, 52, 56].

##### Accessibility of care

Regarding access to professional care, in a Swedish study, adolescents reported that they would like to have a phone number, or a place they could visit, for immediate needs for contact, and information about the way the reception or Health Centre works. There appeared to be a need for several possible routes to accessing professional care: some adolescents preferred contact by phone, some by email, and others wanted to visit in-person. The importance of immediate help being available after making contact was also mentioned [49]. In addition, in an Irish study, the importance of having services that are easily accessible to adolescents and having easy access to mental health professionals at any facility for young people was also highlighted [47].

##### Online services

Adolescents in a New Zealand study highlighted the value of helplines and online forums, because it made them able to connect anonymously with someone, with few consequences for being open and honest [23]. In an Australian study, adolescents were asked to identify preferred sources of potential online support for self-injurious behaviour, using a list of possible sources. The most popular suggestion was for ‘an online service with direct links to professionals in real time via instant messaging’. Regarding online services in general, the following aspects were mentioned as important: guidance, information, reduced isolation, online culture, facilitation of help-seeking, access, and privacy [52].

#### Needs related to schools

Three studies described self-perceived needs of adolescents with suicidal behaviour related to schools [21, 40, 55]. In a UK study, when asked ‘What do you think can be done to help prevent young people from feeling that they want to harm themselves’, many responses of adolescents in a school-based survey with lived experience of self-harm indicated factors concerning school-related issues (35% of 318), such as alleviating the pressure of exams, having someone to talk to about issues apart from the teacher, reducing bullying (e.g., more effective policies) and teachers having more awareness of pupils having emotional problems [21]. In a Brazilian study, adolescents expressed a demand for a responsive and careful dialog, stating that the school (staff) needs support to discuss the topic of self-injury, as adolescents who harm themselves do not speak out [55]. Finally, a study from the US found that, when returning to school after hospitalization for self-harm, adolescents wished for a gradual return to catching up on work and support around work completion [40].

#### Needs related to the general public

Other needs identified by adolescents were related to reducing taboo and stigma around suicidal behaviour [21, 33, 51]. For example, male adolescents in a UK study indicated the importance of breaking the taboo against males seeking help for mental health problems [31]. Another UK study found that, when asked what could be done in the future to help others who self-harm, adolescents emphasized, among other things, the need to increase knowledge about self-harm in the wider population: *“There should be more posters around. You see a lot of things on the television, alcoholics get a lot of help, like ring AA, but there’s no help towards self-harm”* [33]*.* Finally, in a study from Pakistan, adolescents said that they could be encouraged and motivated through use of positive stories from lives of famous people in the media: *“There should be stories of those who wanted to attempt suicide but then found a better way, stories with some message or moral”* [51].

## Discussion

This scoping review was conducted to answer the question ‘What are the self-perceived needs of adolescents with suicidal behaviour?’ An extensive and systematic search through peer-reviewed empirical literature identified 29 relevant studies. These studies differed in terms of purpose, participant demographics, method, and context. Almost half of the studies (13 of 29) were published in the last two years, which indicates that studying young people with lived experience of suicidal behaviour has become a lot more common in recent years and is increasingly considered important.

Unfortunately, there is a paucity of evidence regarding effective interventions for young people with suicidal behaviour [18]. Although the self-perceived needs of adolescents with suicidal behaviour discussed below are important to take into account, they should be seen as possible starting points for prevention, but they are not proven effective interventions in themselves.

### Needs

The identified needs of adolescents with suicidal behaviour covered a wide range of topics. Of the 29 studies reviewed, the majority of needs of adolescents with suicidal behaviour were concentrated on social support and connecting with other people. Supportive connections could be with peers, parents, health care professionals, or any other kind of contacts. Previous research, often quantitative cross-sectional studies, indicates that greater social support is associated with lower suicide risk [58–60]. In addition to these findings, this scoping review showed that among adolescents with suicidal behaviour, there are also self-perceived needs for connection and social support, either preceding self-harm or during the aftercare of these episodes. Contact with peers who had similar experiences was mentioned in a number of studies as an important form of support [43, 47, 53]. Internet forums and social media are increasingly used as places where adolescents find peers who have also self-harmed to communicate about their suicidal feelings and distress [61]. This can be helpful, but at the same time can lead to potential harm in terms of normalizing, triggering, and contagion effects [61–64]. It is important to think about how peer support can be strengthened and used in an effective, safe, and structural way for adolescents who may benefit from this. In addition, a stronger focus is needed on letting adolescents themselves think about beneficial ways peer support can help them.

A substantial part of the included articles described adolescents’ needs for beneficial self-help strategies and personal growth [23, 31, 32, 35, 42, 43, 45, 46, 51, 56]. The majority of these articles emphasize the importance (of having or mastering a diverse set) of self-help coping and distraction techniques for when suicidal tension or the urge to self-harm is high. These topics touch on mental health care where such techniques can be learned, practiced, and discussed. People in close contact with an adolescent with suicidal feelings should also realize the importance of such self-help strategies. Peers or gatekeepers may also be ideally suited to discuss or demonstrate certain techniques with an adolescent. Results of several studies indicate that adolescents often prefer self-help over reliance on clinical services [20, 35]. As a consequence, it seems very important to equip adolescents and their peers with the right tools to maintain their feeling of self-control and independence.

Many other identified needs were focused on the connection with adolescents’ health care providers and the need of adolescents for health care providers to understand them [33, 34, 37, 39, 43, 44, 49, 53, 54, 56]. However, when suicidal tension is high, it is not always easy for health care providers to develop a good, collaborative therapeutic relationship. Also, finding the balance between safety of the patient and patient autonomy is often very challenging. It is, for example, important that health care providers know how and when to use ‘therapeutic risk taking’ (the avoidance of coercive measures to ensure safety in patients who self-harm and instead allow more room for patient autonomy), thereby using shared decision-making to maintain a strong therapeutic relationship to enable recovery [65].

Interestingly, only a small proportion of the identified needs in this review were related to adolescents’ schools or study programs [21, 40, 55]. However, other studies have shown that school-based suicide prevention interventions can be effective [66, 67]. Given that a large proportion of adolescents spend a lot of time in school or college, this topic needs more research in relation to the self-perceived needs of adolescents in such settings.

Finally, a few articles discussed the needs of adolescents in the area of the ‘general public’ [21, 33, 51]. Adolescents remarked on the need to reduce stigma regarding self-harm in society, to increase knowledge about self-harm in the wider population, and for a greater focus on recovery through promotion of stories of well-known/famous people in society overcoming their difficulties [21, 33, 51]. Public awareness campaigns can be used to reduce stigma and taboo in the general public [68–71]. Such campaigns appear to be most effective when they are delivered as part of a broad suicide prevention strategy [68]. The evaluation of a campaign by the Dutch helpline 113 suicide prevention showed that public awareness of the helpline was predominantly in younger people [72]. This specific example shows that it is very important to think about methods to tackle taboo and stigma among all layers of the population and to make information accessible to all target groups.

### Limitations

Overall, there were approximately five times as many female as male participants in the included studies, which is somewhat greater than the sex ratio usually found for young people who self-harm [73]. This is probably explained by the greater participation of females in interview studies on sensitive topics [74, 75]. Furthermore, the studies included in this review were predominantly studies from Anglo countries with Western adolescents. Demographic characteristics regarding sexual orientation or gender identity of the participants were not presented in the studies. Furthermore, we included not only the studies that literally asked about needs, but also studies in which young people with lived experience provided any information about something that was helpful or what they experienced as positive during periods of suicidality. Due to this broad view, the variability in the nature of research focus and aims of the included papers presented a challenge for synthesizing the results of this review. However, we have employed every effort to synthesize the literature in a comprehensive way. Also, for further research, it is important to focus not only on ‘needs’ and thus the positive, helping aspects for the whole picture of prevention in young people with suicidal behaviour, but also on the aspects that have been unhelpful or criticisms that young people have. However, we believe that a focus on needs and on how to fulfill them is important to develop prevention targeted on youth. Although we deliberately chose the age group 10–25 years, age boundaries are arbitrary and the self-perceived needs founded in this review may also be relevant to emerging adults who were older than 25 years. Several studies show this slightly older age group also has self-perceived needs related to, among others, positive relationships and meaningful connections [76–80]. Finally, the reviewed studies varied in quality, but we tried to provide insight into the quality of each study using critical appraisal (see Table 1).

## Conclusions

This scoping review identified studies on the needs of adolescents with suicidal behaviour in peer-reviewed literature. These can broadly be categorized into the following categories of needs: the importance of connecting with other people; adolescents’ self-help strategies and personal growth after self-harm; aspects of mental health care; school; and needs related to society in relation to taboo on suicidal behaviour. Male adolescents with suicidal behaviour were underrepresented as participants in most studies included in this review, so more research into their needs is required. The findings of this study imply that adolescents should be more involved in shaping peer-related suicide prevention. It is important to teach adolescents the right tools and self-help strategies to help them maintain a sense of self-control and independence. More work needs to be done around educating the public about self-harm and suicide prevention and fighting stigma and taboo throughout society. In addition, it is important that health care professionals listen openly and without judgment, that there are multiple routes for getting mental health care, and that online help is available. Also, further research should focus on additional high-risk groups for suicidal behaviour, e.g., non-western adolescents (immigrant and refugee youth), LGBTQ + adolescents, and foster youth but could also focus on a broader group of adolescents, such as those with mental health issues (and without suicidal behaviour). The results of this scoping review may provide leads to develop or strengthen suicide-related prevention taking into account the self-perceived needs of adolescents with suicidal behaviour.

## Supplementary Information

Below is the link to the electronic supplementary material.

Supplementary file1 (DOCX 13 KB)

## Data Availability

The authors declare that the data supporting the findings of this study are available within the paper and its supplementary information files. Should any raw or other data files be needed they are available from the corresponding author upon reasonable request.
