# Supplementary material for: The self-perceived needs of adolescents with suicidal behaviour: a scoping review
Source: Eur Child Adolesc Psychiatry. 2023 Dec 26;34(1):41–67. doi: 10.1007/s00787-023-02342-1 (PMC11805848; doi:10.1007/s00787-023-02342-1)
Supplement: Supplementary file 1 — Supplementary file1 (DOCX 13 KB) [file 787_2023_2342_MOESM1_ESM.docx]

Additional file 1
Search PubMed 18-02-2022

**# Suicide – self harm**

"Self-Injurious Behavior"[Majr] OR "Suicide"[Majr] OR "Self Mutilation"[Majr] OR “suicid*”[ti] OR “self-kill*”[ti] OR “self-poison*”[ti] OR “self-stabb*”[ti] OR “parasuicid*”[ti] OR “self mutilat*”[ti] OR “selfmutilat*”[ti] OR “automutilat*”[ti] OR “auto mutilat*”[ti] OR “Self-Injur*”[ti] OR “Self Harm*”[ti] OR “Self-Destructive Behavio*”[ti] OR “Self cutting”[ti] OR “self directed violence”[ti] OR “self inflicted injur*”[ti] OR “self wounding”[ti] OR “self-inflicted harm”[ti] OR “self-inflicted mutilat*”[ti] OR “self-inflicted wound*”[ti] OR selfharm*[ti] OR “selfinflicted injur*”[ti] OR “selfinflicted wound*”[ti] OR “head banging”[ti] OR “suicid*”[ot] OR “self-kill*”[ot] OR “self-poison*”[ot] OR “self-stabb*”[ot] OR “parasuicid*”[ot] OR “self mutilat*”[ot] OR “selfmutilat*”[ot] OR “automutilat*”[ot] OR “auto mutilat*”[ot] OR “Self-Injur*”[ot] OR “Self Harm*”[ot] OR “Self-Destructive Behavio*”[ot] OR “Self cutting”[ot] OR “self directed violence”[ot] OR “self inflicted injur*”[ot] OR “self wounding”[ot] OR “self-inflicted harm”[ot] OR “self-inflicted mutilat*”[ot] OR “self-inflicted wound*”[ot] OR selfharm*[ot] OR “selfinflicted injur*”[ot] OR “selfinflicted wound*”[ot] OR “head banging”[ot]

**# Adolescents**

"Adolescent"[Mesh] OR "Young Adult"[Mesh] OR Adolescen*[tiab] OR Teens[tiab] OR Teen[tiab] OR Teenager*[tiab] OR Youth[tiab] OR Youths[tiab] OR “young adult*”[tiab] OR “young age”[tiab] OR “young patient*”[tiab] OR “young person*”[tiab] OR “student*”[tiab] OR “young people*”[tiab] OR youngsters [tiab]

**# Needs**

"Health Services Needs and Demand"[MeSH] OR "Needs Assessment"[Mesh] OR Needs[tiab] OR wishes[tiab] OR expectation[tiab] OR expectations[tiab] OR views[tiab] OR perceptions[tiab] OR perception[tiab] OR preference*[tiab] OR “desired care”[tiab] OR demand*[tiab] OR (require*[tiab] AND prevent*[tiab])
